# Challenges and Advances for Genetic Engineering of Non-model Bacteria and Uses in Consolidated Bioprocessing

**DOI:** 10.3389/fmicb.2017.02060

**Published:** 2017-10-24

**Authors:** Qiang Yan, Stephen S. Fong

**Affiliations:** ^1^Department of Chemical and Life Science Engineering, Virginia Commonwealth University, Richmond, VA, United States; ^2^Center for the Study of Biological Complexity, Virginia Commonwealth University, Richmond, VA, United States

**Keywords:** non-model organism, shuttle vector, promoter, genome-scale metabolic models, homologous recombination, CRISPR/Cas9, consolidated bioprocessing

## Abstract

Metabolic diversity in microorganisms can provide the basis for creating novel biochemical products. However, most metabolic engineering projects utilize a handful of established model organisms and thus, a challenge for harnessing the potential of novel microbial functions is the ability to either heterologously express novel genes or directly utilize non-model organisms. Genetic manipulation of non-model microorganisms is still challenging due to organism-specific nuances that hinder universal molecular genetic tools and translatable knowledge of intracellular biochemical pathways and regulatory mechanisms. However, in the past several years, unprecedented progress has been made in synthetic biology, molecular genetics tools development, applications of omics data techniques, and computational tools that can aid in developing non-model hosts in a systematic manner. In this review, we focus on concerns and approaches related to working with non-model microorganisms including developing molecular genetics tools such as shuttle vectors, selectable markers, and expression systems. In addition, we will discuss: (1) current techniques in controlling gene expression (transcriptional/translational level), (2) advances in site-specific genome engineering tools [homologous recombination (HR) and clustered regularly interspaced short palindromic repeats (CRISPR)], and (3) advances in genome-scale metabolic models (GSMMs) in guiding design of non-model species. Application of these principles to metabolic engineering strategies for consolidated bioprocessing (CBP) will be discussed along with some brief comments on foreseeable future prospects.

## Introduction

**FIGURE 1:**
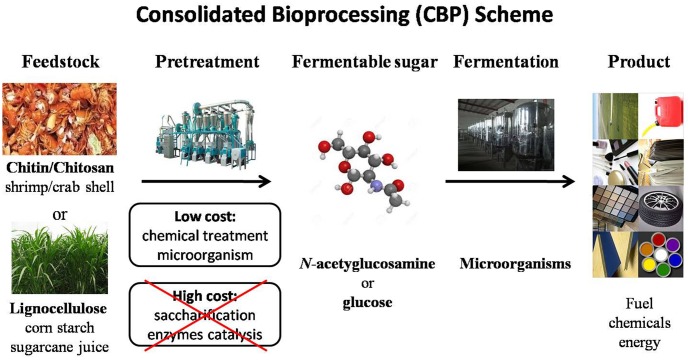
Scheme of consolidated bioprocessing (CBP) from biorenewable feedstock biomass.

With increasing prices of fossil fuel, development of sustainable biorefineries using microorganisms has received great research interest. In particular, one-step conversion (CBP) of naturally renewable biomass such as lignocelluloses has been attractive to researchers in recent years (Lynd et al., 2002, 2005; Xu et al., 2009; Olson et al., 2012). The CBP approach is mainly motivated by three benefits (**Figure 1**). First, it is a sustainable green approach that can significantly reduce greenhouse gas emissions. Second, many renewable feedstocks (lignocelluloses) are readily available, inexpensive resources that can lower material costs. Third, CBP is able to eliminate labor and capital cost of biomass processing by employing a single process step. CBP is widely recognized as the ideal configuration for sustainable, low-cost hydrolysis and fermentation of cellulosic biomass (Lynd et al., 2005; Olson et al., 2012). In principle, a CBP strategy can be applied to produce a broad range of chemicals from natural biomass. It requires degrading recalcitrant biomass substrates into solubilized sugars and metabolic intervention to direct metabolic flux toward desired products at high yield and titer.

Microorganisms natively possess the ability to metabolize different sugars, but the specific sugars and regulation of sugar catabolism vary. Species that are good CBP candidates typically have several characteristics: (1) They express a cohort of genes that synergistically degrade sugar polymers (most organisms metabolize monomeric sugars); (2) They have biochemical capabilities that connect renewable feedstock input to downstream biochemical production pathways (i.e., *Clostridium* sp. harbors native acetone-butanol-ethanol pathways); (3) They are derived from diverse environmental backgrounds (i.e., thermophilic, acidophilic, autotrophic) and possess beneficial attributes (i.e., high alcohol tolerance, temperature tolerance, pH tolerance). However, development of non-model microorganisms is relatively slow and largely hindered by limited molecular genetic tools and lack of knowledge about their complicated metabolic pathways and regulation.

**FIGURE 2:**
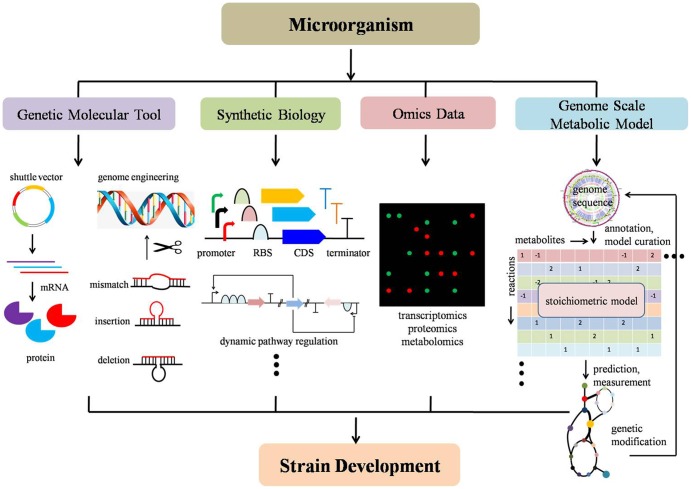
Schematic strategies and techniques for non-model microorganism development. From left to right: (a) genetic molecular tools include building shuttle vectors, genome engineering toolkit, etc; (b) synthetic biology contains modularization of functional biological parts, such as a promoter, ribosome binding site, coding sequence, metabolic biosensors, etc; (c) omics data harbors intracellular high-throughput information, such as transcriptomics, proteomics, metabolomics; (d) genome-scale metabolic model guided strain design to improve target chemical production by predicting a potential gene knock-out, amplification and heterologous expression of foreign pathway genes in a build-test-design manner. Strain development can include improvement targeted chemical production (titer, yield, and rate) or improved sugar utilization (i.e., xylose and pentose).

With recent advances in synthetic biology, molecular tool development, applications of omics data techniques, reconstruction of GSMMs, metabolic flux analysis and genome engineering, non-model microorganisms can now be leveraged to help understand, design, and engineer non-model microorganisms in a systematic manner. Synthetic biology has promoted enthusiasm in understanding and developing novel biological components and has provoked a usage of standard parts into different systems (Registry of Standard Biological Parts^1^). The lower cost of synthesizing DNA enables the redeployment of standard biological parts (i.e., promoters, ribosome binding site, metabolic biosensors) and development of new parts in a plug-and-play fashion (Khalil and Collins, 2010; Way et al., 2014; Yan and Fong, 2016). Continued development of molecular genetic tools (i.e., shuttle vector, reporter gene, expression system) enables successful delivery of foreign constructs, stable replication and efficient expression in non-model microorganisms (Huang et al., 2010). Advances in high-throughput techniques assist in addressing fundamental biological questions as a whole system. Improvement of omics data provides systematic measurements for virtually all types of cellular components in a model organism (Joyce and Palsson, 2006). Advances in metabolic flux analysis offer advantages for accurately determining metabolic fluxes and understanding pathway characteristic (Antoniewicz, 2013; Crown and Antoniewicz, 2013). Novel genome engineering tools provide new genome editing platforms with higher efficiency, more accuracy, and less labor (Wang et al., 2009; Jiang et al., 2013). Advances in GSMMs promote an understanding of cell behavior in a global manner and further provides a powerful method for strain design. Although recent reviews on the development of genetic engineering tools for non-model organisms such as cyanobacteria, microalgae, thermophiles, and fungi can be found (Angermayr et al., 2015; Zeldes et al., 2015; Banerjee et al., 2016; Oliver et al., 2016), it is usually on a case-by-case basis and a comprehensive, systematic, development of non-model strain methodologies from multiple-disciplines perspective needs to be illustrated and summarized (**Figure 2**).

Here, we discuss advances and challenges associated with the development of non-model bacteria as workhorse strains. Specifically, we discuss various aspects of genetic engineering tools and approaches needed to engineer non-model bacteria. Subsequently, an overview of computational tools that can assist in predicting target gene sites to overproduce desired compounds is provided. Finally, a review of state-of-art CBP research is covered as many non-model organisms may have the potential to be developed for use in CBP.

## Design of Shuttle Vector

Development of an efficient, stable, and robust shuttle vector is an important tool for engineering a non-model microorganism, as it is a decisive step for delivering foreign genes into cells, heterologous expression of foreign genes, and is an approach for delivering donor DNA for genome engineering. An effective shuttle vector should possess the following characteristics: (1) It must be able to replicate in the non-model strain or *Escherichia coli* in a stable manner; (2) It must contain efficient and robust selectable markers; (3) It needs to have low homology to the host’s chromosome (in case of allele exchange); (4) It must harbor functional expression elements (i.e., promoter, ribosome binding site).

### Stable Replication

Essential genetic elements for a shuttle vector include a replication origin, expression elements, and a selectable marker. A replication origin is the critical component as it guarantees stable replication of plasmid. Identifying a replication origin in a non-model bacterium typically relies upon two approaches. First, utilize known replication origins (*E. coli*). A variety of *E. coli* replication origins that have different functionalities (copy numbers) have been studied and characterized (Baker and Wickner, 1992). Second, test a suspected replication origin from the non-model organism. One strategy is to construct a shuttle vector containing a native cryptic replication origin in conjugation with an *E. coli* low-copy number replication origin. In this method, shuttle vectors are typically built and propagated in *E. coli*, purified, and transformed into a heterologous host to generate an engineered strain (Chung et al., 2013). One significant concern of such a dual replication origin shuttle vector may be instability either in *E. coli* or in the target hosts. A difference in GC-content between *E. coli* and a Gram-positive host tends to be structurally and segregationally instable in both organisms (Savijoki et al., 1997).

At this point, shuttle vectors containing only one replication origin seem to be superior. Spath et al. (2012) demonstrated that a direct transformation of recombinant expression vectors from PCR/ligation reactions into *Lactobacillus plantarum* yield a compatible transformation efficiency compared to an approach of propagation in *E. coli*. Such an approach has three advantages: (1) It does not pose a requirement of an *E. coli* replication origin; (2) It reduces the amount of bench work; (3) It develops a potential platform for high-throughput screening of a heterogenic pool of mutants. However, the authors also indicated that the transformation efficiency might largely decrease in the presence of restriction modification (RM) systems.

After identifying a replication origin, an expression vector can still be ineffective if it is degraded by intracellular nucleases, especially endonucleases. The active endonucleases in a non-model strain can truncate circular dsDNA at a specific sequence, known as a defensive mechanism, which accounts for the primary barrier for efficient and successful transformation. In nature, methylation at the cutting site is a means for preventing native DNA from host endonuclease. Understanding restriction/methylation (RM) systems on a molecular level (base-to-base) can help develop and improve transformation efficiency. Among *E. coli* RM systems, Dam (G^m^ATC) and Dcm [C^m^C(A/T)GG] methylation systems are two well-characterized methods. For instance, *Clostridium thermocellum* exhibits sequence-specific restriction endonuclease activity (GATC) with little non-specific exonuclease activity. Protection from digestion was observed when plasmids were prepared from a Dam^+^ (G^m^ATC) *E. coli* strain, while plasmids prepared from a Dam^-^ *E. coli* strain were digested by the cell extract cocktail (Klapatch et al., 1996). However, studies also found that additional methylation of plasmid DNA in *E. coli* can inhibit transformation into the target hosts (Kieser and Hopwood, 1990; Macaluso and Mettus, 1991). Recently, Guss et al. (2012) demonstrated that plasmid transformation efficiency was enhanced by 500-fold with the transformation of Dam^+^Dcm^-^ methylated plasmids, rather than Dam^+^Dcm^+^ methylated plasmids, in *C. thermocellum* DSM1313 (Guss et al., 2012). Although the mechanism is still unclear, understanding such RM systems are necessary as they can improve transformation efficiency and shuttle vector stability, especially for Gram-positive bacteria. However, using shuttle vectors still remains challenging in some Gram-positive strains due to the presence of various organism-specific nucleases.

### Selectable Marker

The second essential component for a shuttle vector design is a robust selection system. Antibiotics have been used for selection markers for decades. However, some non-model microorganisms are naturally resistant to common laboratory use of antibiotics. One strategy for selection, based upon antibiotics resistance (ampicillin, kanamycin and chloramphenicol) in a new organism, is to conduct an initial screen to test native resistance by increasing antibiotic concentrations. However, this approach sometimes requires intensive manual labor especially for the expression of genes that hinder the host growth. Less viable approaches include identifying or developing new antibiotic resistance genes or discovery of new antibiotics based upon design-screen-test approaches (Heap et al., 2009). In some circumstances, antibiotics are not a favorable selectable marker due to a specific feature (i.e., thermophiles grown above 70°C might inactivate temperature sensitive antibiotics) or specific considerations for industrial applications (i.e., high cost or not applicable for food-grade application). Two alternative classes of selection markers are dominant markers and complementation markers, shown in **Table 1**. Dominant markers usually confer a new ability to the host strain such as bacteriocin immunity/resistance (Takala and Saris, 2002), heat-shock resistance (El Demerdash et al., 2003), or sugar utilization abilities (Boucher et al., 2002). On the other hand, a complementation selection marker is based on an auxotrophic strain for an essential metabolite (amino acid, DNA/RNA precursor, and sugar), obtained by mutating or deleting the corresponding chromosomal gene, which can be complemented with the plasmid-borne selection gene. Examples of such marker genes include the thymidylate synthase gene (*thyA*) (Sasaki et al., 2004), lactose phosphotransferase gene (*lacF*) (MacCormick et al., 1995) or phosphor-β-galactosidase gene (*lacG*) (Platteeuw et al., 1996), and alanine racemase gene (*alr*) (Nguyen et al., 2011).

**Table 1:** Antibiotic-free selection markers and their resources.

Marker type	Selection marker	Selection marker protein	GenBank ID	Reference
**Complementation marker**				
Amino acid auxotroph	Glycine	Serine hydroxylmethyl transferase (GlyA)	EIQ69691	Vidal et al., 2008
Amino acid auxotroph	Alanine	Alanine racemase (Alr)	CDN27234	Nguyen et al., 2011
Cofactor auxotroph	NAD	Quinolinic acid phosphoribosyltransferase (QAPRTase)	BAE76041	Dong et al., 2010
DNA precursor auxotroph	Thymidine	Thymidylate synthase (ThyA)	E12778	Sasaki et al., 2004
RNA precursor auxotroph	Uracil	Orotidine-5′-phosphate decarboxylase (PyrF)	KFU53019	Schneider et al., 2005
Sugar auxotroph	Lactose	Lactose phosphotransferase (LacF)	AAD45618	MacCormick et al., 1995
Sugar auxotroph	Lactose	Phosphor-β-galactosidase (LacG)	ART26567	Cranenburgh et al., 2004
**Dominant marker**				
Bacteriocin immunity/resistance	Lactacin F	Lactacin F immunity protein operon (Laf)	M57961	Allison and Klaenhammer, 1996
Bacteriocin immunity/resistance	Nisin	Nisin immunity lipoprotein (NisI)	Z18947	Takala and Saris, 2002
Heat-shock resistance	Temperature	Small heat shock protein (Shsp)	AJ242476	El Demerdash et al., 2003
Sugar utilization	Melibiose	Alpha-galactosidase (Aga)	AAO26321	Boucher et al., 2002


### Identification and Characterization Expression System

Promoters are key tools in controlling gene expression since they initiate gene transcription. Promoter regions can be hard to predict in a non-model organism. Therefore, libraries of well-characterized promoters are often only available in well-established organisms. Thus, transcription of foreign genes or activation of cryptic endogenous clusters is one main obstacle for heterologous gene expression or for understanding cryptic novel endogenous cluster functions, respectively. With the advent of high-throughput sequencing technology (mRNA-seq), gene expression levels can be determined with low cost, reliable results, and high efficiency according to the intracellular transcriptomics data. Accordingly, promoters can be rationally designed based on transcription level and tested by using reporter proteins (i.e., GFP).

Recently, systematic identification of constitutive promoters in different microorganisms has been demonstrated as a useful, efficient, and robust platform in *Saccharomyces cerevisiae*, *C. thermocellum*, as well as *Streptomyces* sp. (Sun et al., 2012; Li et al., 2015; Luo et al., 2015; Olson et al., 2015). In order to obtain a library of constitutive promoters, genome-scale transcriptomics data is ranked and compared under various culture conditions or under different time series. Promoter strength can be measured by both protein expression level (GFP) and mRNA level (real-time PCR). Such a panel of constitutive promoters can facilitate precise and quantitative control over gene expression that can potentially be applied to improve microbial chemical production by promoter engineering (Sun et al., 2012; Olson et al., 2015). A strong constitutive promoter is capable of triggering expression of some cryptic clusters, resulting in the discovery of novel natural products and the study of a genetic regulation system (Li et al., 2015; Luo et al., 2015).

## Tuning Gene Expression

In addition to activating native cryptic gene clusters or expressing foreign genes using native constitutive promoters, tuning gene expression levels are also key components in genetic modification of microorganisms. In this review, tuning gene expression level mainly refers to controlling a chromosomal functional gene expression by either downregulation through introducing non-native tools or upregulation through reprogramming the controlling parts.

### Downregulation

Gene downregulation is an effective approach to study gene/protein function or inactivate certain metabolic pathways in non-model hosts. It is sometimes superior to direct gene knock-out mainly because it can avoid the pitfalls of lethal mutations or complete inhibition of protein production. Gene downregulation can be achieved by introducing an exogenous complementary single strand RNA (asRNA) or CRISPR/dCas9 that inactivates at the translational or transcriptional level, respectively.

### Antisense RNA

Antisense RNA has been widely used for downregulating gene expression in many microorganisms. Although the mechanism of asRNA action is not fully understood, asRNA have been successfully applied in non-model organisms, such as *Clostridium* sp. (Desai and Papoutsakis, 1999; Tummala et al., 2003) and *Lactococcus lactis* (Sybesma et al., 2004). Such a method has obvious advantages: First, it can be an alternative approach especially for genes that are essential for cell growth. Second, asRNA can be induced conditionally to reduce gene expression without competing for resources with cell growth (metabolic burden). Third, asRNA can be designed for multiplex genome engineering to repress multiple genes simultaneously (Desai and Papoutsakis, 1999; Yang Y. et al., 2015). However, this approach still requires careful design for the asRNA structure and length before experimental implementation and optimization of the asRNA concentration since it is correlated with the inhibition efficiency. Furthermore, the majority of various intracellular RNA pools have the potential to hinder the efficiency of asRNA (Song et al., 2015).

### CRISPR/dCas9

Recently, a catalytically inactive dead-Cas9 (dCas9) was constructed by introducing the D10A and H840A mutations into each nuclease domain, which removed the nuclease activity while maintaining its ability to bind to the target site (Jinek et al., 2012). This modified and simplified CRISPR/dCas9-mediated transcription repression system was successfully used for gene expression regulation in model strains (Qi et al., 2013; Cress et al., 2016). Compared to the asRNA-based system, which regulates gene expression at the translation level, the CRISPR/dCas9 system controls gene expression at the transcription level. Moreover, the CRISPR/dCas9 system uses exogenous parts that are orthogonal to those in host organism and has less chance of competing with the native regulatory systems. Details regarding the expression and efficiency concerns when applying the CRISPR/Cas9 system in non-model hosts are discussed in the genome engineering section.

### Upregulation

Upregulation of a gene expression from a single copy number level can be beneficial to the host without introducing an exogenous part. Upregulation can be accomplished either by increasing transcriptional rate through addition of an UP element or by accelerating translational initiation rate through reprogramming a stronger RBS sequence.

### UP Element

Upstream elements, a consensus AT-rich sequences located upstream of the -35 promoter region, have been reported as a gene enhancer of transcription since they can alter the interaction between RNAP and promoters by additional binding with the RNAP alpha subunit C-terminal domain (αCTD). UP elements have been reported to enhance bacterial promoter strength in *E. coli* and *Bacillus subtilis* (Frisby and Zuber, 1991; Meng et al., 2001), and recently have been applied in metabolic engineering applications with various successes (Elmore et al., 2017; Yan and Fong, 2017). A novel feature of UP elements was observed to decrease gene expression noise by applying them to *E. coli* constitutive promoters (Yan and Fong, 2017). In the study, a further mechanistic insight between gene expression and *in vitro* RNAP and promoter interaction was found that a decent expression level only occurs at a moderate binding affinity. Thus, UP elements can potentially be used as a simple module to enhance gene expression, with several merits. First, it can facilitate promoter engineering by inserting an UP element to each core promoter by a short ssDNA; Second, multiplex genome engineering can be applied by designing site-specific ssDNAs. Third, UP elements can, potentially, stringently control gene expression by reducing basal leaky expression for inducible promoters.

### Ribosomal Binding Site

Besides the transcriptional level gene upregulation, a gene expression level can also be elevated by carefully tuning its TIRs, namely by design of RBS sequence. The RBS Calculator is a computational program that enables both a *de novo* design of the RBS sequence at a certain TIR (from 0.001 to 100,000) and prediction of an RBS sequence and calculation of the TIR in a bacterial genome (Salis et al., 2009). In the model, the hybridization between an mRNA and 16S rRNA and the interactions between the 30S complex and an mRNA were taken into account to quantify a TIR. Such a model has been applied to non-model hosts for many applications, including manipulating proteins expression level (Pogrebnyakov et al., 2017), optimizing synthetic metabolic pathways (Temme et al., 2012), and predicting TIRs across a genome (Salis, 2011). Temme et al. (2012) demonstrated the capability of utilizing synthetic parts (T7 promoter and RBS) to control 20 nitrogen fixation genes expression in *Klebsiella oxytoca*. Elmore et al. (2017) developed a chromosomal insertion method for functional gene expression in *Pseudomonas putida* KT2440, which allowed a panel of promoter library (-35/-10 variants) characterization. The author also showed that the expression was further tuned by 2-fold with redesign of RBS and insertion of UP-elements (Elmore et al., 2017).

## Development of Genome Engineering Toolbox

### Homologous Recombination

Homologous recombination is one of most commonly used site-specific genome engineering approaches. HR can facilitate an in-frame deletion by an endogenous exonuclease (e.g., RecA in *E. coli*) in conjugation with an exogenous DNA into non-model hosts (Radding et al., 1983; Deng and Fong, 2010). However, the native HR system efficiency is relatively low, and successful operations require a careful consideration of conditions (i.e., transformation efficiency, competent cell, selection) (Deng and Fong, 2010). The HR efficiency can also be improved by introducing a heterologous recombination system, including a Cre recombinase (Khodakaramian et al., 2006), a Cre-like tyrosine recombinase (Dre) (Herrmann et al., 2012), a Flp recombinase (Fedoryshyn et al., 2008) and a bacteriophage lambda recombinase (Kim et al., 2008; Lindenkamp et al., 2012). Reviews regarding these approaches can be found recently (Fogg et al., 2014; Deng and Zhang, 2017).

Recently, HR was applied for multiplex genome engineering using ssDNA, an approach named MAGE (Wang et al., 2009, 2012a,b; Isaacs et al., 2011). In this method, a *mutS* mutated *E. coli* harbors lambda-Red genes. It is believed that ssDNAs prefer to target the lagging strand rather than the leading strand due to the much higher efficiency (more than 50-fold). The lambda-Red HR facilitates the ssDNA’s targeting at the lagging strand of the replicating genome to introduce mutations. In the MAGE application paper, the authors demonstrated engineering 20 gene RBSs for optimizing the 1-deoxy-D-xylose-5-phosphate (DXP) pathway to produce lycopene (Wang et al., 2009). In order to overcome a common barrier for fast and efficient selection of a genomic mutant after MAGE, development of methods for co-selection markers along with target sites proved to be able to enhance the recombination efficiency of MAGE to greater than 70% (Wang et al., 2012b). Nevertheless, some improvements still need to be considered to carry on: (1) MAGE is limited to integrate large genes (>1 kb) since it depends on ssDNAs; (2) it is still a challenge to widely use this methodology in other non-model bacteria.

### Clustered Regulatory Interspaced Short Palindromic Repeats (CRISPR)

A CRISPR/Cas9 system is composed of a clustered set of Cas genes and the signature CRISPR array (Deveau et al., 2010; Horvath and Barrangou, 2010; Terns and Terns, 2011). The Cas9 gene is translated into an endonuclease, whereas the repeated spacer array is transcribed into a long precursor and subsequently processed to generate small crRNAs that direct the endonuclease to cleave dsDNA at specific target sequences (protospacers) (Hale et al., 2009; Makarova et al., 2011). The PAMs that are located immediately downstream of the protospacer is another essential element responsible for directing cleavage of DNA (Deltcheva et al., 2011; Hatoum-Asian et al., 2011).

Such CRISPR/Cas9 systems genome editing tools have been programmed and developed in a wide range of organisms (DiCarlo et al., 2013; Feng et al., 2013; Jiang et al., 2013; Wang H. et al., 2013). In bacteria, the CRISPR/Cas9-mediated genome editing tools rely on introducing two foreign components: Cas9 and a crRNA-trancrRNA duplex or a single guide RNA (sgRNA). **Table 2** summarizes current strategies of developing CRISPR/Cas9 for genome editing in non-model bacteria. Excellent reviews can also be found describing general consideration and design when harnessing the CRISPR/Cas9 system in bacteria (Hsu et al., 2014; Selle and Barrangou, 2015; Luo et al., 2016; Mougiakos et al., 2016).

**Table 2:** Development of CRISPR/Cas9 expression systems for genome engineering in bacteria.

Host	Endonuclease	Promoters	Guiding RNA	Function	Repair mechanism	Reference
*C. beijerinckii*	Cas9	spoIIE	sgRNA	Deletion	HR with double-stranded template	Wang et al., 2015
*C. cellulolyticum*	Cas9 (D10A)	P4 synthetic promoter	sgRNA	Deletion/insertion	NHEJ with double-stranded template	Xu et al., 2015
*C. saccharoperbutylacetonicum*	Cas9	P_J23119_	gRNA	Deletion	HR with double-stranded template	Wang et al., 2017
*E. coli*	Cas9	*S. pneumonia cas9* promoter	tracrRNA-crRNA^a^	Deletion	HR with double-stranded template	Jiang et al., 2013
*E. coli*	Cas9	J23119	sgRNA	Insertion/deletion	Lambda-Red HR and single-stranded template	Jiang et al., 2015
*L. reuteri*	Cas9	*S. pneumonia cas9* promoter	tracrRNA-crRNA	Deletion	HR with single-stranded template	Oh and van Pijkeren, 2014
*S. pneumonia*	Cas9	*S. pneumonia cas9* promoter	tracrRNA-crRNA	Deletion	HR with double-stranded template	Jiang et al., 2013
*Streptomyces* sp.	Cas9	rpsLp/rpsLp/gapdhp	sgRNA	Deletion	HR with double-stranded template	Cobb et al., 2015
*Streptomyces* sp.	Cas9	PtipA/J23119	sgRNA	Deletion	HR with double-stranded template	Huang et al., 2015
*Streptomyces* sp.	Cas9	PtipA	sgRNA	Deletion	NHEJ/HR with double-stranded template	Tong et al., 2015


*^a^Dual expression of tracrRNA and crRNA.*

The high efficiency of CRISPR/Cas9 genome editing tool can reach an efficiency up to 100% due to its ability to select out edited cells from non-edited background cells. The first successful CRISPR/Cas9 systems genome editing in bacteria was published in Jiang et al. (2013). The authors combined Cas9 with a CRISPR array and a trancrRNA to generate targeted genome editing in both *Streptococcus pneumonia* and in *E. coli*. A separate oligonucleotide whose sequence contained a mutated PAM sequence that can prevent recognition and cleavage from the endonuclease after a repair from HR. Thus, the CRISPR/Cas9 system can not only make defined site mutation but also served as ‘cleanup’ role by eliminating cells that does not undergo recombination. After the initial publication in *S. pneumonia* and *E. coli*, applications of CRISPR/Cas9-mediated genome editing have been demonstrated in non-model bacteria, such as *Lactobacillus reuteri* (Oh and van Pijkeren, 2014), *C. beijerinckii* (Wang et al., 2015), *C. cellulolyticum* (Xu et al., 2015), *Streptomyces* sp. (Cobb et al., 2015; Tong et al., 2015). Chimeric sgRNA designs were subsequently investigated instead of the dual-RNA expression of a tracrRNA and crRNA system. CRISPR/Cas9-based multiplex genome targets were also investigated (Cobb et al., 2015).

Applying this technology for non-model bacteria faces challenges and requires careful consideration of several factors. First, the wide range of microbial diversity poses challenges for basic genetic manipulation. For example, heterologous expression of Cas9 in non-model bacteria may be hindered due to a lack of efficient approaches such as transformation, plasmid replication, and an ability for gene expression. From this point of view, exploring the native machinery system seems to be a superior strategy by precluding heterologous expression of Cas9. And experimental evidence was reported in some *Clostridium* species that the native CRISPR/Cas9 system (Type I-B) performed higher editing efficiency (100% versus 25%) than the heterologous Type II CRISPR/Cas9 system (Pyne et al., 2016). Second, understanding the PAM sequence is useful for design as PAM and seed sequences are essential for recognition and activity for the gene target. Third, although a 20-nt guide sequence of the sgRNA is believed to guide the Cas9 to the target sequence, potential off-target cleavage activity could still occur on DNA sequences with three to five base pair mismatches. Many computational softwares can be readily found for designing sgRNA with a goal of improving accuracy (Doench et al., 2014, 2016; Moreno-Meteos et al., 2015). Fourth, since the CRISPR/Cas9 system is sometimes lethal to bacteria due to introduced dsDNA breaks, the reparation of DSB is not possible. Thus, a dsDNA or an ssDNA was supplied to facilitate HR or NHEJ as a selection tool against the non-edited background cells (Wang et al., 2015, 2016). HR can be achieved either using its native HR system (i.e., RecBCD in *E. coli*) (Wang et al., 2015; Cobb et al., 2015) or overexpression of the lambda-Red recombination system (Jiang et al., 2015). Another strategy is to use mutated Cas9 (D10A or H840A), which are believed to reduce the lethality of cleaving by nicking the Cas9 target site without introducing a double-stranded break. For example, Xu et al. (2015) introduced a mutated Cas9 (D10A) and sgRNA as well as an dsDNA template in the same vector resulted in efficiency up to 95%, while the normal Cas9 did not yield any colonies. To date, there are few studies implementing CRISPR/Cas9 genome editing using the NHEJ repair strategy. Tong et al. (2015) expressed LigD along with sgRNA and Cas9 to complete the NHEJ repair system in *S. coelicolor*, and demonstrated the feasibility of introducing NHEJ in bacteria for CRISPR/Cas9 genome editing (Tong et al., 2015).

## Genome-Scale Metabolic Model Based Strain Improvement

With the advent of modern genome sequencing, GSMMs have been developed as a powerful and indispensable tool to study and predict microorganism metabolism, physiology, and phenotype. In general, GSMM is based upon a stoichiometric mathematical model through integrating genome annotation, biochemical knowledge and every reaction information in a target organism. By bridging the gap between genome-based biochemical information and metabolic phenotype in a principled manner, GSMM offers an overall perspective on the metabolism of whole cells. A general four-step protocol for GSMM reconstruction was summarized as draft reconstruction, manual curation, conversion to a computational format, and network evaluation and validation (Thiele and Palsson, 2010).

GSMM based *in silico* design for predicting key genetic targets to guide experimental implementation in microbial chemical production has been given with various successes. It can be accomplished by predicting the flux intervention through gene knock-out (Segre et al., 2002; Burgard et al., 2003; Shlomi et al., 2005), upregulation/downregulation (Pharkya and Maranas, 2006; Choi et al., 2010; Kim and Reed, 2010; Ranganathan et al., 2010), and heterologous gene expression of foreign pathways (Doerks et al., 2002; **Table 3**).

**Table 3:** Function of computational tools in identifying target gene for strain design.

Methodology	Description	Reference
BioPathway predictor	Identification non-native pathway by known chemical reactions and analysis according to various restrictions (e.g., maximum theoretical yield, pathway length, thermodynamic feasibility, etc).	Yim et al., 2011
BNICE	Identification novel pathways using a “generalized enzyme reaction” and evaluation by pathway length and thermodynamics of chemical formation	Hatzimanikatis et al., 2004
FSEOF	Identification gene amplification targets in response to an enforced objective flux of product formation on a genome-scale basis	Choi et al., 2010
MOMA	Prediction a metabolic phenotype of gene knock-out strain by minimizing a distance in flux space	Segre et al., 2002
OptForce	Prediction increase/decrease of a flux value to meet a pre-specific overproduction target	Ranganathan et al., 2010
OptGene	Prediction gene deletion targets to overproduce a desired product	Patil et al., 2005
OptKnock	Prediction gene deletion that maximizes target pathway flux	Burgard et al., 2003
OptORF	Prediction gene deletion or amplification targets by integrating transcriptional regulatory networks and metabolic networks	Kim and Reed, 2010
OptReg	Prediction deletion or amplification to overproduce a target product	Pharkya and Maranas, 2006
OptStrain	Prediction deletion or identification heterologous expression gene target to aid microbial strain design (pathway balancing, maximum product yield, optimal substrate and microbial host)	Pharkya et al., 2004
ROOM	Prediction knock-out strain metabolic fluxes at steady state by minimizing the number of significant flux changes.	Shlomi et al., 2005


The first computational-aided strain design program, Optknock, was developed for simulating gene deletion by employing a bi-level optimization program to seek reaction knock-out targets that would yield overproduction of a desired compound while maintaining optimal growth (Burgard et al., 2003). Soon thereafter, a series of OptKnock-derived programs have been developed for various gene manipulations other than knock-out, such as OptGene (Patil et al., 2005), OptForce (Ranganathan et al., 2010), OptORF (Kim and Reed, 2010), OptReg (Pharkya and Maranas, 2006), and OptStrain (Doerks et al., 2002). MOMA is a computational tool that calculates the changes to reaction fluxes when a gene is deleted. A result in an optimal flux state is seen with MOMA which is closest resembles a given flux distribution observed in a wild-type strain (Segre et al., 2002). Similar to MOMA, ROOM also predicts putative flux distributions after gene deletions by minimizing the number of significant flux changes (Shlomi et al., 2005).

The second aspect for *in silico* strain design involves predicting and simulating target gene amplification. This approach can be reached by computational identification of the flux changes (following by gene-protein-reaction) in response to the pathway toward the target chemical and can be experimentally achievable by increasing the expression of related genes. For example, the method named FSEOF is aiming to identify gene amplification targets by scanning the changes of all the metabolic fluxes in response to the enhancement of the flux toward the target compound (Choi et al., 2010). Some OptKnock derived programs such as OptReg, OptORF, and OptForce can be used to predict gene amplifications and to investigate upregulation/downregulation of genes in target organisms.

Constraint-based modeling can account for heterologous expression if the gene and function are known. There are fewer options available to computational approach to develop *de novo* pathways. BNICE was able to identify novel pathways using a “generalized enzyme reaction” according to the third level classification. Each pathway was examined on a basis of thermodynamics for chemical formation and pathway length (Hatzimanikatis et al., 2004). Biopathway predictor allowed identification of non-native pathway based on a transformation of functional groups by known chemical reactions. The pathway can be further analyzed by various restrictions (maximum theoretical yield, pathway length, thermodynamic feasibilities) (Yim et al., 2011). Based on the pathways the specific model optimized, and certain strain modifications, a proof-of-concept was demonstrated with 18 g/L of 1,4-butanediol, a non-natural chemical in *E. coli*.

In order to comprehend strain phenotypes changes in response to gene modifications novel conceptual models are necessary and need to be developed. An approach named ME-model was developed in *Thermotoga maritima* by considering the production and degradation of a cell’s macromolecular machinery (transcription, translation and the dilution of gene products to daughter cells) (Lerman et al., 2012). Such models enable new predictive capabilities: first, ME-models allow a direct prediction of transcriptome and proteome based upon a gene sequence. Second, cellular costs can be considered since each cellular composition is associated with gene products. For example, longer pathways have a higher enzyme production cost than shorter pathways, and this cost can be predicted in the ME-model. Further improvement of the ME-model extended to protein translocation in the cell membrane, all enzyme structures, and transcriptional regulation. A second conceptual advance in GSMM is modularity of simulation strategies. A whole-cell model of *Mycoplasma genitalium* was constructed with 525 genes and 28 modules (Karr et al., 2012). The authors developed multiple modularized subsystems using different modeling approaches (e.g., Boolean statements, constraint-based, probabilistic, ordinary differential equations) to dynamically simulate each process at discrete time points. The model was used to explore protein-DNA association rates, the relationship between DNA replication and initiation, and to direct experimental elucidation of kinetic parameters.

These computational methods can be readily deployed to study non-model organisms if their genome has been sequenced. When developing the initial model, care must be taken to look at potentially novel functions that cannot be adequately described by homology. In addition, non-model organisms may use familiar biochemical pathways in a novel map (novel metabolic cycles and regulation), so experimental testing of a new computational model is always beneficial.

## Integrate Omics Data to GSMM for Strain Design

With the significant decrease of the cost of data generation and analysis, omics data can be implemented for an organism in a very short time. Integration of the omics data to the GSMM can help improve the accuracy of prediction for the metabolic fluxes. One approach to deploy the omics data to the GSMM is called “the switch approach,” which controls on/off reaction fluxes based on threshold expression levels (lower bound and upper bound) (Hyduke et al., 2013; Saha et al., 2014). The key parameter is to choose the arbitrary cutoffs for gene expression because the method assumes that one reaction is considered “off” if the gene/protein level is below the threshold. Further criterions or submodels were developed to re-enable lowly expressed genes associated with low flux enzyme activities in the cases where model fails to simulate the global phenotype (Akesson et al., 2004; Shlomi et al., 2008). The other approach is known as “the valve approach,” which controls on/off reaction fluxes based on relative gene/protein expressions instead of the absolute gene/protein expression levels. For instance, Colijn et al. (2008) utilized expression levels for a gene product as linear adjustments to allow regulation of flux with associated activities. The algorithm was applied to integrate transcriptomic data to simulate *Mycobacterium tuberculosis* metabolism and the model was able to identify 7 of 8 known inhibitors and predict several candidate inhibitors for potential therapeutics.

Thus far, several applications have been applied using the omics data integrated GSMM for production of desired chemicals (Gowen and Fong, 2010; Vanee et al., 2014). Compared to the FBA based approach for strain design, which usually optimize a maximum cell biomass or chemical production as an objective function, integrating omics data can guide the model in a real-time manner. At the meantime, the model is expected to provide more insights to pinpoint the main carbon flux, the complicated regulation and signaling system to improve chemical titer (Dai and Nielsen, 2015). For instance, Becker et al. (2011) coupled a GSMM with the metabolimics to target for L-lysine production in *Corynebacterium glutamicum* (from zero to hero), where the final engineered strain produce a titer of 120 g/L L-lysine with a high yield of 0.55 g/g glucose (Becker et al., 2011).

## Non-Model Bacteria for CBP Scheme

A number of studies have explored the benefits of developing CBP based on the engineering of the native microbial chassis. The most developed CBP applications focus on using cellulose and cellulolytic organisms with goals of: (1) efficiently degrading and hydrolyzing natural biomass into available sugar; and (2) reinforcing metabolic fluxes toward a desired compound. However, due to the recalcitrant nature of cellulosic biomass, degradation and hydrolysis on untreated biomass is a slow and difficult process.

### Decomposing Cellulose by Cellulase Regulation

Genetic engineering approaches can improve microbial cellulolytic degradation through strategies such as increasing secretion enzyme quantity, improving an enzymatic property, investigating cellulosome regulatory mechanism and enhancing cellular growth on specific biomass (Liambertz et al., 2014; Book et al., 2016). Recently, the roles, activities, and regulatory mechanisms controlling expression of different cellulases were studied, and found to be an effective approach to facilitate cellulose degradation or increase cellulase production (Deng and Fong, 2010; Wang S. et al., 2013; Aghcheh et al., 2014; Häkkinen et al., 2014). The improved cellulase activity can reach from a 2- to 16.69-fold increase after either relieving repression or activating production. An example of combining these approaches was accomplished by carefully blocking intracellular inducer hydrolysis (deletion *bgl2*), increasing the activator (overexpression *clrB*) and relieving the repression (deletion *creA*) simultaneously in *Penicillium oxalicum*. The optimized strain enabled an increase of cellulase activities from 10- to 50-fold (Yao et al., 2015).

### Metabolic Engineering Strategies

The other main challenge relies upon reinforcing flux toward desired chemicals to increase titer, yield, and productivity. In terms of various pathways for biofuel production as a CBP scheme (Liao et al., 2016), every opportunity to increase product titer, yield or productivity was explored. It always starts with creating/overexpression of desired pathway genes, followed by deletion of competitive pathways to drain precursors, products and cofactors. Production titers can be further improved through beneficial metabolic strategies, including eliminating toxic intermediates, enhancing driving force for pathway flux, and prolonging the cellular production phase. Specifically, these metabolic engineering strategies can be technically achieved by approaches such as promoter engineering, pathway modification by global mutation and selection strategies, cofactor regeneration, knock-out competitive pathway and co-culture. A summary of recent publications on metabolic engineering strategies for native CBP microorganisms is shown in **Table 4**.

**Table 4:** Recent metabolic strategies using native CBP bacteria for biofuel production.

Type of strategy	Product	Titer	Host	Reference
Co-culture	Butanol	7.9 g/L	*C. acetobutylicum*	Nakayama et al., 2011
			*C. saccharoperbutylacetonicum*	
Co-culture	Acetone	2.64 g/L	*C. cellulovorans,*	Wen et al., 2014
	Butanol	8.30 g/L	*C. beijerinckii*	
	Ethanol	0.87 g/L		
Co-culture	Acetone	4.25 g/L	*C. cellulovorans,*	Wen et al., 2016
	Butanol	11.5 g/L	*C. beijerinckii*	
	Ethanol	6.37 g/L		
Cofactor engineering	Ethanol	40 mM	*C. thermocellum*	Deng et al., 2013
Cofactor engineering	*n*-butanol	0.85 g/L	*Thermoanaerobacterium saccharolyticum*	Bhandiwad et al., 2014
Cofactor engineering	Ethanol	1.60 g/L	*C. cellulovorans*	Yang X. et al., 2015
	*n*-butanol	1.42 g/L		
Cofactor engineering	Ethanol	5.1 g/L	*C. thermocellum*	Lo et al., 2017
Elimination competitive pathway	*n*-butanol	10.0 g/L	*C. tyrobutyricum*	Yu et al., 2011
Elimination competitive pathway	Ethanol	12.8 mM	*Caldicellulosiruptor bescii*	Chung et al., 2014
Elimination competitive pathway	Ethanol	37 mM	*C. thermocellum*	Biswas et al., 2015
Elimination competitive pathway	Ethanol	73.4 mM	*C. thermocellum*	Papanek et al., 2015
Promoter engineering	Isobutanol	5.4 g/L	*C. thermocellum*	Lin et al., 2015
RBS engineering	Acetone	21.9 g/L	*C. acetobutylicum*	Yang et al., 2016
	Butanol			
	Ethanol			
Pathway modification	1-butanol	29.9 mg/L	*Synechococcus elongates* PCC1942	Lan and Liao, 2012
Pathway modification	Acetone		*C. beijerinckii*	Xiao et al., 2012
	Butanol	16.91 g/L		
	Ethanol			
Pathway modification	Isopropanol	14.63 g/L	*C. acetobutylicum*	Dusseaux et al., 2013
	Butanol	4.75 g/L		
	Ethanol	1.01 g/L		
Pathway modification	Acetone	5.4 g/L	*C. acetobutylicum*	Bormann et al., 2014
	Butanol	16.9 g/L		
	Ethanol	3.6 g/L		
Pathway modification	1-butanol	–	*Methylobacterium extorquens*	Hu and Lidstrom, 2014
Pathway modification	*n*-butanol	15.7 g/L	*C. tyrobutyricum*	Yu et al., 2015


*^a^Not reported.*

Promoter engineering can facilitate to enhance production titer by adjusting the metabolic burden found during cloning and by reinforcing the rate-limiting step through pathway balancing. For example, in order to get a stable and robust construct for propagating in *E. coli* and for expression in *C. acetobutylicum*, Lin et al. (2015) identified and characterized 21 *C. acetobutylicum* native promoters to drive isobutanol synthetic operons and tested isobutanol production. This study provided a solution of stable and robust expression for a heterologous pathway in non-model microorganisms (Lin et al., 2015). In order to prolong cellular ABE production phase, an exogenous four-step biotin synthetic operon was introduced in *C. acetobutylicum* ATCC 824 and the gene cluster expression level was tuned by engineering the RBS region. The optimized engineered strain can be boosted to produce solvent titer 21.9 g/L and productivity 0.30 g/L h (Yang et al., 2016).

Pathway modification is another useful strategy for increasing alcohol production by identifying the rate-limiting step and pulling the flux by switching a higher performance enzyme. In the CoA-dependent 1-butanol pathway in *C. acetobutylicum*, NADH and acetyl-CoA pool serve as driving forces. Shen et al. (2011) demonstrated that modification of the native pathway by switching enzymes (use NADH without coupling ferredoxin/flavoproteins as reducing source) or higher specific activity enzyme can improve 1-butanol production. In order to increase the ratio of butanol to acetone without the formation of additional ethanol, Bormann et al. (2014) demonstrated that utilizing a more butanol-specific aldehyde and alcohol dehydrogenase enabled selectively increase butanol titers while maintaining acetone production. The molar ratio of butanol to acetone after optimization improved from 2.04 to 2.45. Pathway modification can also be extended to introducing a non-native pathway to facilitate more flux toward a desired product. Due to a thermodynamically unfavorable formation of crotonyl-CoA through acetyl-CoA in the first step of 1-butanol formation, an alternative route through malonyl-CoA synthesis was introduced in *Synechococcus elongatus* PCC7942. Although the resulting pathway resulted in more ATP consumption, the optimized strain enabled direct 1-butanol production (29.9 mg/L) after photosynthesis while the acetyl-CoA dependent pathway barely detected 1-butanol (6.5 mg/L) (Lan and Liao, 2012).

Cofactor engineering is an important strategy for alcohol production in the CoA-dependent pathway where NADH/NADPH is the main supply of reduced cofactors. Disrupting competitive pathways (i.e., formate, hydrogen) can contribute to increasing alcohol production by increasing electron supply (Biswas et al., 2015). Papanek et al. (2015) reported that deletion of genes involved in acetate, lactate, formate, and most of the hydrogen production in a single strain can yield a maximum ethanol titer of 73.4 mM. Understanding electron transfer and cofactor supply can also favor improving alcohol production since NADH is the main electron donor for alcohol formation. Deng et al. (2013) demonstrated an endogenous three-step pathway (called malate shunt) converting phosphoenolpyruvate to pyruvate, which contains an NADH-linked malate dehydrogenase. By disrupting the malate shunt pathway genes coupled with expressing a pyruvate kinase, ethanol production can increase 3 to 3.25-fold (Deng et al., 2013). Lo et al. (2017) investigated different electron supply resource’s effects on fuel production and identified that an NAD^+^ oxidoreductase is the main supply for NADH regeneration. The results showed that overexpression of *rnf* genes resulted in an increase in ethanol production of about 30% (Lo et al., 2017).

Recently, co-culture has been developed as an effective metabolic engineering strategy that can construct and modulate each organism’s expression system and pathway in parallel so that the time required for making the product would be substantially reduced (Zhou et al., 2015). In CBP schematic fuel production, an artificial consortium can create a symbiotic relationship to produce fuel in a synergistic manner. Nakayama et al. (2011) developed a cooperator-cooperator consortium by a cellulolytic strain and a butanol-producing strain. The cellulolytic strain secreted butyric acid that can induce butanol production in the butanol-producing strain. The co-cultured system produced 7.9 g/L butanol from 4% Avicel after 9 days of incubation (Nakayama et al., 2011). In order to ferment a variety of sugars (i.e., hexose, pentose, xylose) other than cellulose, the co-cultured system can be developed based on a strain’s metabolic capability. Wen et al. (2014) constructed a co-culture of *C. cellulovorans* and *C. beijerinckii*. Under an optimized condition, the co-culture produced 11.9 g/L of solvents from 68.6 g/L alkali pretreated corn cobs (Wen et al., 2014). In the following study, the authors genetically modified the cellulolytic strain to pull more flux toward butyrate production and the solvent-producing strain to enhance organic acids reassimilation and pentose utilization. The engineered consortium was shown to produce 22.1 g/L of solvents from 83.2 g/L lignocelluloses hydrolysate (Wen et al., 2016).

## Future Perspective

Although improvements have been made in CBP over last 5 years, issues are still present regardless native strains or recombinant strains: (1) Microbial biofuel production titer is unsatisfactory to meet an industrial scale (i.e., majority of CBP publication study use substrate concentrations of less than 10 g/L); (2) Few publications have been reported using direct untreated raw materials; (3) Progress with hosts for the native CBP microorganism is slower because tools are less developed.

We believe that research related to CBP should be focused on several aspects. First, strain improvements will focus on industry-scale conditions (i.e., high cellulose concentration). A recent interesting study was conducted to fit the industry-level at 100 g/L cellulose (Holwerda et al., 2014). It was found that the strain growth ceased at about half of the substrate had been consumed, while fermentation continued till substrate was completely depleted. A wide range of fermentative products not seen at lower substrate concentration was produced; potentially raising an issue that the modified strain at low substrate concentration may not perform ideally to the industry-scale requirement. Thus, a further in-depth analysis of how an organism will behave at industrially relevant conditions should be conducted and strain improvements and process engineering techniques may need to be developed accordingly.

Second, cost should be taken into account on an industry-level basis (i.e., using untreated raw biomass). A rough PubMed literature search utilizing the keyword of “consolidated bioprocessing” revealed a total of 254 publications, while less than 40 publications used untreated or chemically/mechanically pre-treated cellulose/hemicellulose biomass, less than 10 publications used untreated cellulose/hemicelluloses biomass. Fermentation data of using untreated raw biomass should be valuable for guiding strain improvements during chemical production. Strain development coupled with fermentation strategies should also be investigated based on a ‘real-world’ condition.

Third, production titers should be explicitly linked to molecular mechanism. Such considerations could help understand the mechanism of intracellular regulation mechanism and help to minimize the metabolic burden cost introducing during strain genetic modification (Wu et al., 2016). GSMMs are capable of quantification on a molecular level being able to demonstrate regulation mechanism and should be helpful to enhance the titer of production. For example, a reassimilation from organic acid to solvents occurs in *Clostridium* sp. during biofuel fermentation. Such complexity and nature of the systematic process is still unclear, hindering understanding and optimization of solvent production. GSMMs can provide an insightful prediction on a system level. Liao et al. (2015) used a modulated GSMM framework that combines metabolic reactions, gene regulation and environmental cues (pH) to simulate solvent and acid production during acetone-butanol-ethanol fermentation. The model simulation fitted well with experimental data at various key genes deletion strains and fermentation pH conditions. Dash et al. investigated the *C. acetobutylicum* response to butyric acid and butanol stress on a genetic regulation basis using GSMM and CoreReg algorithm. The model predicted a core regulation at arginine and amino acid metabolism at butanol stress while a core regulation at arginine and pyrimidine metabolism at butyric acid stress (Dash et al., 2014). Another example for strain design was established for *C. thermocellum* DSM 1313. After reconstruction, the model predicted that ATP is essential for cell growth on cellulosome, and it investigated the cellodextrin length on cell growth. Furthermore, using the model can assist to provide potential genetic modification strategies for target production (Thompson et al., 2016). Future work is expected to focus on utilizing those well-trained models to predict potential strain improvement target and validate by experimental implementation instead of matching with existing experimental data.

Inspired by CBP lessons from cellulolytic biomass, the CBP can be conceptually extended to bioconversion of low cost natural biomass or wastes into value-added products without introducing enzyme hydrolysis. In other words, any organism that harbors the capability of utilizing low-cost naturally biomass or waste will ideally be genetically modified and engineered to create a potential route for value-added products. For instance, as the world’s second most abundant polymer, chitin/chitosan occurring as a main component in seafood wastes (i.e., shrimp, crab, lobster shells) (Yan and Fong, 2015). Annually, such organic marine waste products pose a potential issue to the world and society: disposal has an associated high capital cost (e.g., $150/ton in Australia while dried shrimp cost $100–120/ton and estimated 1.5 million tons in Southeast Asia alone) (Dash et al., 2014). Applications of CBP by conversion of these marine wastes into value-added products can not only reduce the expensive cost for disposal but also can create a sustainable way to create more value (Yan et al., 2017), the potential value of shells for the chemical industry is being ignored. A “shell biorefinery” project was proposed with a multimillion-dollar funding to establish the first processing pipeline in the next 5 years (Yan and Chen, 2015). With the advances in the above-mentioned techniques, development of CBP application using microorganisms to target a chitin-based substrate should provide an alternative approach.

## Conclusion

Metabolic engineering of non-model microorganisms have recently received an enormous amount of research interest due to their high diversity of properties and capabilities of these organisms. However, progress on developing non-model strains is slow, mainly due to the lack of developed genetic engineering tools and their less well-defined systems. In this review, we discussed methods and considerations for developing molecular genetic tools. Methods for controlling gene expression are evaluated both for downregulation and upregulation. Current genome engineering methodologies and design concerns were provided. GSMM computational frameworks were summarized as a tool for strain design. A stare-of-art CBP application was updated with metabolic engineering strategies. Future prospects were proposed: (1) more research should focus on industry-level condition (i.e., high cellulose concentration, untreated raw material); (2) a combination of GSMM-based strain design and experimental implementation is expected; (3) an extension of CBP application to other raw biomass should be developed.

## Author Contributions

All authors listed, have made a substantial, direct and intellectual contribution to the work. QY wrote and revised this manuscript; SF revised this manuscript.

## Conflict of Interest Statement

The authors declare that the research was conducted in the absence of any commercial or financial relationships that could be construed as a potential conflict of interest.

## Footnotes

1 http://parts.igem.org

## Abbreviations

asRNA: antisense RNA
BNICE: biochemical network integrated computational explorer
αCTD: C-terminal domain of the α subunit of RNAP
Cas: CRISPR-associated
CBP: consolidated bioprocessing
CRISPR: clustered regularly interspaced short palindromic repeats
crRNA: CRISPR RNA
dCas9: dead Cas9
dsDNA: double-stranded DNA
FBA: flux balance analysis
FSEOF: flux scanning based on enforced objective flux
GFP: green fluorescent protein
GSMM: genome-scale metabolic model
HR: homologous recombination
MAGE: multiplex automated genome engineering
ME-model: metabolism and macromolecular expression model
MOMA: minimization of metabolic adjustment
NHEJ: non-homologous end joining
PAM: protospacer adjacent motifs
RBS: ribosomal binding site
RM system: restriction/methylation system
RNAP: RNA polymerase
ROOM: regulatory on/off minimization
sgRNA: single-guide RNA
ssDNA: single-stranded DNA
TIR: translation initiation rate
tracrRNA: *trans*-acting crRNA
UP element: upstream element
